# Effectiveness of diabetes self-management education and support interventions on glycemic levels among people living with type 2 diabetes in the WHO African Region: a Systematic Review and meta-analysis

**DOI:** 10.3389/fcdhc.2025.1554524

**Published:** 2025-06-03

**Authors:** Yimer Seid Yimer, Adamu Addissie, Eshetu Girma Kidane, Ahmed Reja, Abdurezak Ahmed Abdela, Ahmed Ali Ahmed

**Affiliations:** ^1^ Department of Epidemiology and Biostatistics, School of Public Health, Addis Ababa University, Addis Ababa, Ethiopia; ^2^ African Population and Health Research Center, Nairobi, Kenya; ^3^ Department of Internal Medicine, School of Medicine, Addis Ababa University, Addis Ababa, Ethiopia

**Keywords:** type 2 diabetes, diabetes self-management education and support, WHO Africa Region, systematic review, meta-analysis

## Abstract

**Background:**

For successful glycemic control, diabetes control requires a comprehensive management plan in which patients are educated and supported to make informed decisions about diet, exercise, weight control, blood glucose monitoring, taking medication, and regular screening for complications. Current evidence on the effectiveness of diabetes self-management education and support (D-SMES) interventions on blood glucose control is mixed, with some studies pointing to significant glycemic control benefits, whereas others have shown no significant benefits.

**Objective:**

This systematic review and meta-analysis (SRMA) was conducted to evaluate the effectiveness of D-SMES interventions compared with usual care in controlling blood glucose levels among people living with type 2 diabetes (T2DM) in the World Health Organization (WHO) Africa Region and to describe the core components of D-SMES interventions.

**Methods:**

We performed a SRMA of D-SMES interventions for managing T2DM in the WHO Africa Region. We searched PubMed, CINAHL, the Cochrane Central Register of Controlled Trials (CCRCT), and Google Scholar from inception to May 5, 2025, for studies that were randomized control trials that reported glycated hemoglobin (HbA1c) or fasting blood sugar (FBS) as outcome measures and were delivered to adults with T2DM. The methodological quality of the included studies was assessed via the Cochrane risk of bias tool (RoB2). Random effects model meta-analysis was used to estimate the population average pooled standard mean difference (Hedges’ g) for HbA1c with 95% CIs.

**Results:**

We screened the title/abstract records of 350 studies, of which 19 studies with a total of 3759 participants (1866 in the D-SMES group and 1893 in the usual care group) were included in the meta-analysis of HbA1c. The meta-analysis revealed a significant overall effect of D-SMES interventions on HbA1c among people living with T2DM in the WHO African Region (SMD = -0.468 with a 95% CI of -0.658 to -0.279, I2 = 85.5%). nine of the nineteen included studies reported significant effects. We would expect that in some 95% of all populations comparable to those in the analysis, the true effect size would fall between -1.27 and 0.34 (prediction interval). Of the 19 included studies, 15 had a low risk of bias, two had high risk, and two raised some concerns based on the Cochrane RoB 2 tool.

**Conclusions:**

Diabetes self-management education and support interventions are moderately effective in controlling blood glucose levels in T2DM patients within the WHO African region.

**Systematic Review Registration:**

https://www.crd.york.ac.uk/PROSPERO, identifier CRD42022375732.

## Background

Diabetes mellitus (DM) is a chronic metabolic condition characterized by hyperglycemia resulting from defects in insulin secretion, insulin action, or both, can leads to severe damage to the heart, blood vessels, eyes, kidneys, and nerves over time, if not treated properly (1, 2).

Type 2 diabetes mellitus (T2DM) is the most common type of diabetes, accounting for approximately 90% of all diabetes cases. It is generally characterized by insulin resistance, where the body does not fully respond to insulin. T2DM is associated with a family history of diabetes, overweight or obesity, an unhealthy diet, physical inactivity, increased age, and high blood pressure (1).

In the IDF Africa Region, one in 20 (25 million) adults aged 20-79 years were living with diabetes in 2024. This number is predicted to increase by 142% to 60 million by 2050. Four in Five (73%) people living with diabetes are undiagnosed. Diabetes was responsible for 216,000 deaths in 2024 in Africa (1).

The progression of diabetes and its complications can be prevented through strict glycemic control. Epidemiological analysis of the UK Prospective Diabetes Study (UKPDS) data revealed that for every 1% reduction in HbA1c, the relative risk for microvascular complications decreased by 37%, that for diabetes-related deaths decreased by 21%, and that for myocardial infarction decreased by 14% (3).

To achieve or maintain the target of glycemic control, diabetes requires a comprehensive management plan in which patients are educated and supported to make informed decisions about diet, exercise, weight control, blood glucose monitoring, medication adherence, and regular screening for complications. In this context, the WHO Global Action Plan on Control of NCDs (4) aims to reduce the burden of NCDs by promoting healthy lifestyles; reducing common risk factors; providing integrated evidence-based, innovative, and cost-effective public health and clinical interventions; and suggesting strategic interventions for decentralizing and integrating NCD services and preventing NCD risk factors into primary health care (PHC) through task shifting.

Self-management is the set of tasks individuals undertake to help them live with one or more long-term conditions (such as eating healthily, being more physically active, and controlling their blood glucose to manage their diabetes). D-SMES interventions are aimed at improving self-care behaviors. A useful framework for defining the scope of self-management interventions is provided by the taxonomy proposed in the Practical Systematic Reviews in Self-Management Support (PRISMS) (5). The PRISMS taxonomy comprises 14 distinct components that may be delivered directly to people with long-term conditions (LTCs) and/or their caregivers to support self-management. These include: 1) information about conditions or management; 2) information about available resources; 3) provision of or agreement on specific clinical action plans and/or rescue medication; 4) regular clinical review; 5) monitoring of conditions with feedback; 6) practical support with taking medication or doing recommended behaviors); 7) provision of equipment; 8) provision of easy access to advise or support when needed; 9) training or rehearsal to communicate with health care professionals; 10) training or rehearsal for everyday activities; 11) training or rehearsal for practical self-management activities; 12) training or rehearsal for psychological strategies; 13) social support; and 14) lifestyle advice and support. Self-management support is typically multifaceted, and the expectation is that several (although not necessarily all) of the PRISMS components may be present in interventions. Several studies, mainly conducted in high-income countries, provide considerable evidence supporting D-SMES interventions as cost-effective and clinically effective in the prevention and management of T2DM by reducing body weight and improving glucose control (6–10).

Current evidence on the effects of D-SMES interventions on blood glucose control is mixed, with some studies pointing to significant glycemic benefits (11–16), whereas others have shown no significant benefits (17–19).

According to Dube and colleagues, D-SMES interventions in most African countries are limited in scope, content, and consistency, and it is unclear how patients from Sub-Saharan Africa (SSA) manage their diabetes (20). Although a systematic review conducted in 2018 among people living with T2DM in SSA revealed that the provision of structured D-SMES was effective in improving patients’ behaviors and health outcomes (21), the finding was based on limited data (only six out of the 43 reviewed studies were based on D-SMES interventions). One recent scoping review on D-SMES in the WHO African Region (22) is available, but it includes different research designs, such as randomized controlled trials, quasi-experimental studies, mixed methods, and observational cohort studies.

On the other hand, we did not find any ongoing SRMA considered to investigate the effectiveness of D-SMES interventions on glycemic levels among peoples living with T2DM.

Therefore, the aim of this SRMA is to determine the effect of D-SMES interventions on glycemic levels in adults living with T2DM in the WHO African Region.

In our systematic review and meta-analysis, we aimed to answer the following two questions (1): Are D-SMES interventions, compared with usual care, effective in improving blood glucose levels among adult patients with T2DM in the WHO African Region? (2) What are the core components of D-SMES interventions, specifically in relation to intervention characteristics for the management of T2DM (method, context of delivery, provider, strategy, intervention duration, and intensity)?

## Methods

This SRMA was conducted and reported in accordance with the Preferred Reporting Items for Systematic Reviews and Meta-Analyses (PRISMA) (23). The PRISMA 2020 checklist for reporting systematic reviews and meta-analyses is presented in **Additional File 1**. This review was registered prospectively on the International Prospective Register of Systematic Reviews (PROSPERO 2022: CRD42022375732).

### PICO eligibility criteria

#### Population

This SRMA considered studies carried out among T2DM patients living in the WHO African Region.

#### Interventions

We included studies assessing any D-SMES interventions for T2DM that matched at least one of the fourteen categories of the Practical Reviews in Self-Management Support (PRISMS) taxonomy (5). There were no inclusion limits on the frequency, duration, or delivery mode of the intervention. No restrictions were applied regarding the year of publication.

#### Comparators

Studies comparing D-SMES interventions with standard or usual diabetes care were included in this SRMA. Standard or usual diabetes care includes routine medical consultation and follow-up from healthcare providers on the basis of the lifestyle and self-care treatment algorithms recommended by the country’s NCD management guidelines.

#### Outcome

Studies that assessed HbA1c as an outcome measure were included. When average blood glucose (ABG) levels were reported, we used a formula proposed by Nathan DM et al. (24) to convert ABG into HbA1c.

#### Types of studies

Only randomized controlled trials (RCTs) at community or outpatient health facility settings were included in this SRMA. Cluster RCTs were included if the unit of analysis was at the patient level.

### Exclusion criteria

We excluded studies with the following characteristics: type 1 diabetes, gestational diabetes, studies outside of the WHO African region, study reports written in languages other than English, studies in which the outcome was not reported (either HbA1c or ABG), review protocols, review articles, SRMAs, editorials, qualitative, mixed methods, quasi-experimental, pre-post, and observational studies.

### Search strategy

A systematic electronic literature search was conducted to retrieve eligible studies from PubMed (PubMed Central, MEDLINE), CINAHL, CCRCT, and Google Scholar. In addition, we further searched the reference lists of all the included papers and previous reviews. The search was conducted from inception until May 5, 2025.

A combination of search terms was used. The search strategy was developed via the Yale Mesh Analyser on the basis of the PubMed identification (PMID) number of the ten initially identified articles. Accordingly, we developed and constructed the following combined search terms for each PICO criterion (**Table 1**).

**Table 1:** Combined search terms based on the PICO criterion to evaluate the effect of D-SMES interventions on HbA1c among T2DM patients in the WHO African Region.

PICO criterion	Search term	Boolean operators
Population	“Diabet* mellitus” OR “diabet*” OR “noninsulin” OR “hyperglycemia” OR “type 2*” OR “T2DM” OR “T2D” OR “type 2 diabet*” OR “type II diabet*” OR “non-insulin dependent diabet*” OR “NIDD”	AND
Intervention	“Diabet* self-management education” OR “diabet* self-management educational program” OR “diabet* self-care education” OR “nurse-led diabet* self-management education” OR “diabet* self-management intervention” OR “diabet* self-management intervention program” OR “diabet* self-care intervention” OR “nurse-led diabet* self-management intervention” OR “lifestyle behavior change intervention” OR “lifestyle behavior change intervention” OR “structured lifestyle diabet* education program” OR “self-management education” OR “self-support education” OR “self-care education”	AND
Comparator	“Usual clinical care” OR “usual diabetes care” OR “usual care” OR “conventional care alone” OR “conventional education” OR “standard of care” OR “treatment-as-usual” OR “treatment as usual”	AND
Outcome	“glyc*” OR “HbA1c” OR “A1C” OR “FBG” OR “fasting blood glucose” OR “blood glucose” OR “clinical outcomes” OR “long-term glyc* control” OR “optimal glyc* control” OR “glucose control” OR “clinical status”	AND
Study Design	“randomi*” OR “Controlled Clinical Trial” OR “Controlled Trial” OR “Clinical Trial” OR “experimental clinical trials” OR “interventional study” OR “clustered randomized trial” OR “randomized clustered trial” OR “clustered randomized controlled trial”	AND
Context	“Developing Countries” OR “Resource-Limited Countries” OR “Africa” OR “low and middle-income countries” OR “poor resource settings” OR “African region”	AND

### Study selection

All identified citations were exported to EndNote reference management software to manage duplications, and then two independent reviewers (YS and EG) searched and screened the titles and abstracts of the remaining articles against the inclusion criteria. We subsequently searched for the full texts of the eligible articles.

### Risk of bias (quality) assessment

To assess how thoroughly studies addressed potential bias in their design, conduct, and analysis, two independent reviewers (YS and EG) evaluated the methodological quality of the selected articles via Version 2 of the Cochrane risk-of-bias tool for RCTs (RoB 2) (25). Each article was assessed across the five domains of the RoB 2 tool: Domain 1 (risk of bias from randomization), Domain 2 (risk of bias from deviations in intended interventions), Domain 3 (missing outcome data), Domain 4 (risk of bias in outcome measurement), and Domain 5 (risk of bias in selection of reported results). The articles were rated as ‘Low’ or ‘High’ risk of bias or ‘Some concerns’ for each domain. The overall risk-of-bias judgment was defined as follows: a low risk of bias indicated that the study was assessed as having low risk in all domains for that result; some concerns indicated that at least one domain raised concerns, but the study was not at high risk in any domain; a high risk of bias indicated that the study had high risk in at least one domain or had concerns in multiple domains that substantially reduced confidence in the result. After the quality assessments were completed, the reviewers met to discuss and resolve any discrepancies. The findings from this evaluation were then used to guide the synthesis and interpretation of the study results.

### Data extraction

A data extraction tool, Microsoft Excel, was used to extract data, including the characteristics of the study, characteristics of D-SMES interventions, and effect size data. Two reviewers (YSY and EGK) independently extracted the data to ensure data reliability and trustworthiness. When differences occurred, a conclusion was reached through consensus. We present the data extracted from the included studies in **Additional File 2**.

### Assessment of heterogeneity

We assessed heterogeneity by reviewing the characteristics of the included studies. We also reviewed the forest plot of the included studies to determine whether the confidence intervals for the results of individual studies had poor overlap. In addition, Cochran’s Q test was used to determine whether there were differences between studies or if the variation observed was due to chance. A low P value of <0.10 in the Q test was considered to provide evidence of variation in effect estimates beyond chance. To determine what proportion of observed variance was real and the variance of true effect sizes, we used I^2^ statistics and the Tau square, respectively. To determine how much the true effect varies, we estimated the prediction interval. The true effect size is the effect we would see if we could enroll the entire population of the study. Furthermore, we explored the source of heterogeneity by conducting sensitivity analysis, subgroup analysis, and meta-regression.

Sensitivity analysis was conducted to ensure that the results were not overly influenced by any study. Subgroup analyses were conducted via mixed effects analysis (a random effects model was used to combine studies within each subgroup, and a fixed effect model was used to combine subgroups) to explore whether intervention characteristics such as setting, intervention modality, intervention content, intervention implementation strategy, application of behavior change theory, duration of intervention might explain some of the variation.

Meta-regression was conducted to explore the effects of multiple factors (characteristics of studies) simultaneously on the pooled effect estimate and to discuss the proportion of variance explained by each factor. The regression coefficient obtained from the meta-regression analysis was used to describe how the SMD changes with a unit increase in the explanatory variable.

### Data synthesis

First, we described the characteristics of the included studies in terms of the different study and intervention characteristics and the risks of bias. A meta-analysis was subsequently conducted for the outcome (HbA1c) via Comprehensive Meta-analysis Software Version 3. The postintervention SMD (Hedges’ g) of HbA1c was pooled via random effects models. Cohen suggested that SMDs of 0.2, 0.5, and 0.8 are considered small, medium, and large effect sizes, respectively (26). We chose the random effects model for three reasons. First, it allowed us to take into account the study’s variance when assigning weights to each study. Second, it allowed us to assess the dispersion in effect size across studies (assess the study variance). Third, this model could allow us to generalize to comparable studies from the studies included in the analysis. Furthermore, the random effects model was intended to adjust for both explained and unexplained heterogeneity.

### Assessment of publication bias

We tested the presence of small-study effects via one of the regression-based tests: the Egger test and the Begg rank correlation test and performed a trim-and-fill analysis. The main idea behind these tests was to determine whether there was a statistically significant association between the effect sizes and their measures of precision.

## Results

### Literature selection

**Figure 1:**
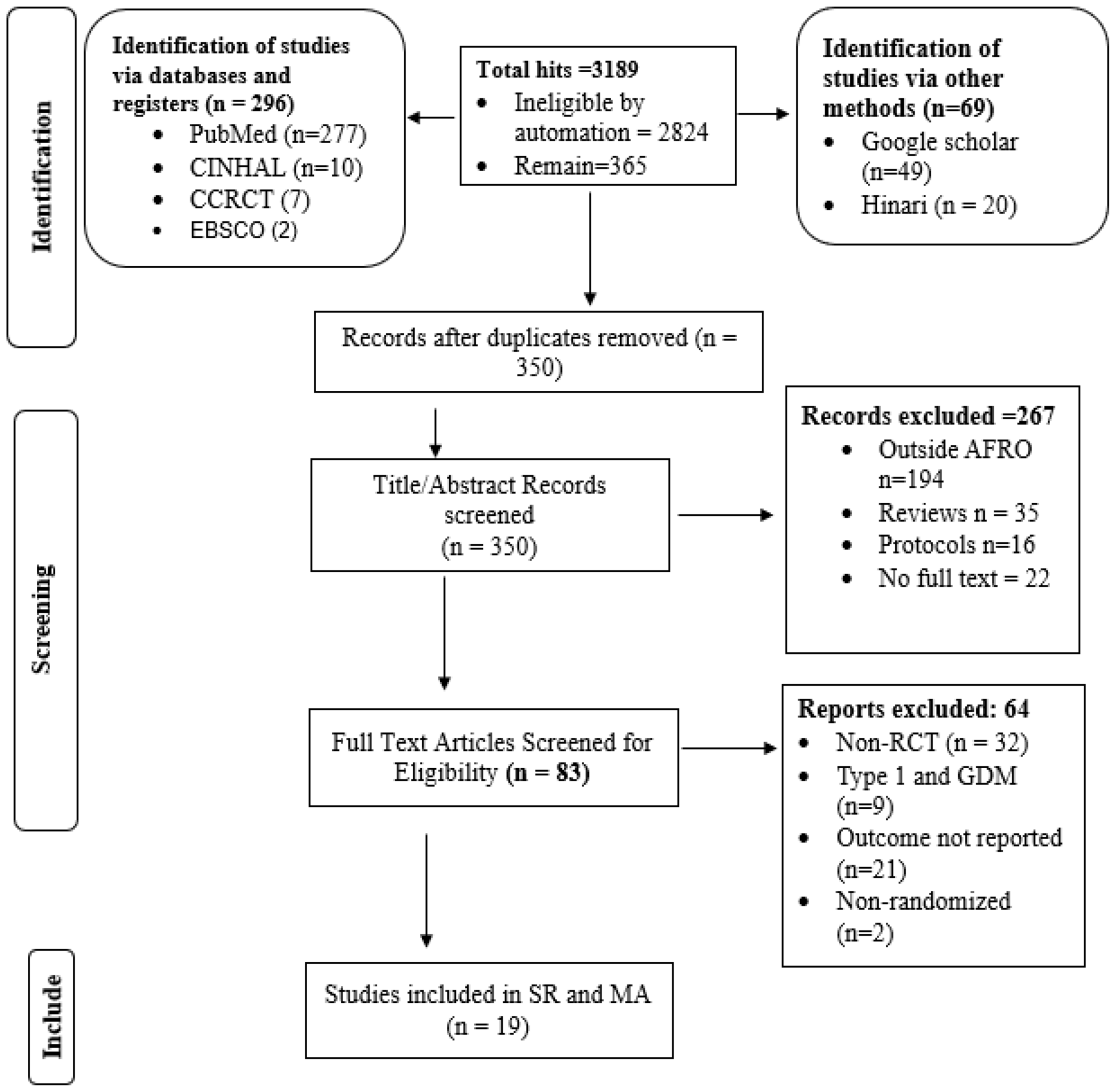
PRISMA 2020 study flow diagram for selecting studies to evaluate the effect of D-SMES interventions on HbA1c among people living with T2DM in the WHO African Region.

Among the 3189 search results, 2824 records were marked as ineligible by automation tools (advanced search options, including age group, sex, place, article type, language etc.). A total of 365 articles were exported to EndNote, and 15 duplicate articles were excluded. Following review by title and abstract, 83 articles progressed to full-text review. Among these studies, 64 were excluded for not meeting the inclusion criteria, including 32 non-RCTs, 9 studies on type 1 diabetes and GDM, 21 studies with no reported outcomes, and 2 nonrandomized studies. The remaining 19 trials were included in this review. The detailed process is illustrated in **Figure 1**.

### Study characteristics

In terms of where the interventions were delivered, ten of the 19 included trials were conducted at hospital outpatient settings (11, 13–15, 27–32), five were conducted at primary health care facilities (12, 17, 18, 33, 34), and the remaining four interventions were community-based (16, 35–37). Five of the studies were conducted in South Africa (12, 18, 28, 29, 37), three in Nigeria (14, 15, 30) and others in different countries of Africa. The total sample size was 3759 (1866 in the D-SMES group and 1893 in the control group), with individual study participants ranging from 41 (33) to 1022 (37). The average duration of follow-up in the included studies was 7.5 months, with a minimum of two months and a maximum of 12 months.

The most commonly investigated outcome measures were HbA1c (n = 18 studies), and only one study (14) reported average blood glucose (ABG) values. **Table 2** provides a detailed account of all the study characteristics.

**Table 2:** Characteristics of the included studies evaluating the effect of D-SMES interventions on HbA1c among patients with T2DM in the WHO Africa Region.

Study name	Year of publication	Country	Setting	Duration of follow-up	SMES* group sample size	Usual Care group sample size	Effect size (SMD*)
Assah et al. (35)	2015	Cameroon	Community	6 months	96	96	-1.225
Debussche et al. (16)	2018	Mali	Community	12 months	76	75	-0.594
Essien et al. (15)	2017	Nigeria	Hospital outpatient	6 months	53	51	-1.121
Gathu et al. (17)	2018	Kenya	PHC	6 months	51	45	-0.274
Hailu et al. (19)	2018	Ethiopia	Hospital outpatient	9 months	78	64	-0.173
Yan et al. (33)	2014	Mozambique	PHC	3 months	31	10	0.000
Muchiri et al. (12)	2015	South Africa	PHC	12 months	41	41	-2.000
Muchiri et al. (29)	2021	South Africa	Hospital outpatient	12 months	39	38	-0.050
Ojieabu et al. (14)	2017	Nigeria	Hospital outpatient	4 months	75	75	-0.302
Mash et al. (18)	2014	South Africa	PHC	12 months	391	475	-0.189
Ng’ang’a et al. (34)	2022	Rwanda	PHC	6 months	38	35	-0.466
Asante et al. (27)	2020	Ghana	Hospital outpatient	3 months	30	30	-0.631
Van Rooijen et al. (28)	2010	South Africa	Hospital outpatient	12 months	23	20	-0.197
Amendezo et al. (13)	2017	Rwanda	Hospital outpatient	12 months	115	108	-0.447
David EA et al. (30)	2021	Nigeria	Hospital outpatient clinic	3 months	54	54	-0.647
Farmer et al. (37)	2021	South Africa and Malawi	Community Based	12 months	510	512	-0.042
Lamptey R et al. (31)	2023	Ghana	Hospital outpatient clinic	3 months	79	80	0.000
Thuita et al. (32)	2020	Kenya	Hospital outpatient clinic	6 months	48	46	-0.386
Diriba DC et al. (36)	2023	Ethiopia	Community Based	2 months	38	38	-0.049

*SMES, Self-Management Education and Support; *SMD, Standardized Mean Difference.

### D-SMES intervention characteristics

The majority of the included studies (n = 14) were group-based D-SMES interventions; the remaining five studies were individual-based. In five studies, interventions were delivered by health care providers (11, 13, 15, 31, 34), peer educators/supporters (n = 3) (16, 27, 35), dieticians (n = 2) (12, 29), research teams (n = 6) (28, 30, 32, 33, 36, 37), diabetes educators (n = 1) (17), pharmacists (n = 1) (14), and health promoters (n = 1) (18).

In terms of intervention content, the majority of the included studies (n = 15) focused on multiple components of the D-SMES intervention, including diabetes education and counselling, dietary intervention, physical exercise, and blood glucose monitoring. Two studies focused only on dietary interventions (12, 29), one study focused on physical exercise (33), and one study focused on self-management of blood glucose (34).

The majority of the included studies (n = 16) used multifaceted intervention strategies, including two or more of the following: education, counselling, goal setting, problem solving, experience sharing, reminders, follow-up and supervision, and educational and diagnostic material provision. The remaining three studies used a discrete type of implementation strategy, such as supervised exercise (33), mobile phone follow-up (27), and diabetes education (14). Seven of the 19 studies used theoretical models to bring about the desired behavior change, including social–cognitive theory (16, 29, 36), empowerment theory (17, 28), and motivational interviewing principles (18, 37). **Additional file 3** provides an overview of D-SMES intervention characteristics.

### Risk of bias (quality) assessment

Out of the 19 studies included, 15 were assessed as having a “low risk” of bias across all domains according to the Cochrane RoB 2 tool. Two studies were judged to have a “high risk” of bias (14, 35), while the remaining two raised “some concerns.” (18, 33). The randomization method was described adequately in 15 trials. A major source of bias identified across all trials was that the participants and implementation providers were not blinded. The detailed results of the quality assessment based on the Cochrane RoB 2 tool are presented in **Additional File 4**.

### Effects of D-SMES interventions on blood glucose levels (HbA1c)

#### The mean effect size

**Figure 2:**
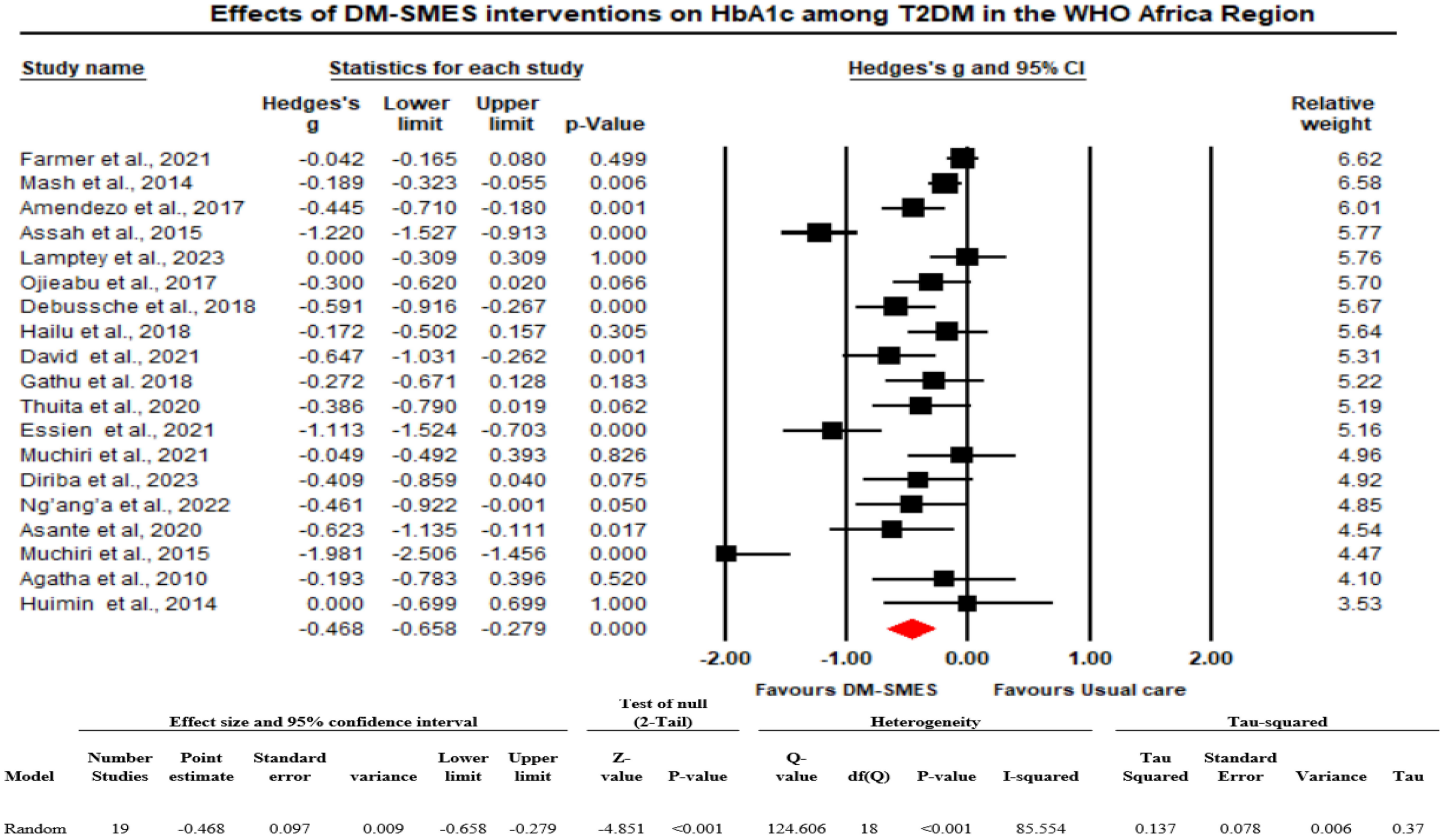
Effects of D-SMES interventions on HbA1c among people living with T2DM in the WHO Africa Region.

The analysis is based on 19 studies. The effect size index is the standardized difference in means (Hedges’ g). On average, in populations that are comparable to those in the analysis, the intervention decreased HbA1c by approximately 0.468 standard deviations (the mean SMD is -0.468 with a 95% CI of -0.658 to -0.279), with a prediction interval ranging from -1.27 to 0.34. **Figure 2** shows the effect of D-SMES interventions on HbA1c.

##### How much does the effect size vary across studies?

The Q statistic provides a test of the null hypothesis that all studies in the analysis shared a common effect size. The Q value was 124.606 with 18 degrees of freedom, and the p value was <0.001. Using a criterion of 0.10, we rejected the null hypothesis that the true effect size was the same in all those studies and concluded that the true effect size varies across studies. The I^2^ statistic (proportion of real variance) is 85.5%, which tells us that some 85.5% of the variance in observed effects reflects variance in true effects rather than sampling error. Tau squared (the variance of true effect sizes) is 0.137, and Tau, the standard deviation (SD) of true effect sizes, is 0.370. If we assume that the true effects are normally distributed, we can estimate that the prediction interval is -1.27 to 0.34. We would expect that in some 95% of all populations comparable to those in the analysis, the true effect size would fall between -1.27 and 0.34 (**Figure 2**).

### Sensitivity analysis

To ensure that the results were not overly influenced by an outlier (i.e., how much of an impact each study has on the analysis). First, we checked how much weight was assigned to each study. Accordingly, the relative weight of each study was not more than 7%, and we also observed that almost every study had at least 3% weight, which shows that no one study dominated the analysis. Second, we sorted the studies by effect size and carried out the analysis with only one study removed. Accordingly, the mean effect size never moves to the right or to the left, and the p values are <0.001, which shows that the basic conclusion remains unchanged when any one study is removed.

### Publication bias/small-study effect

To assess publication bias, we conducted both Egger’s regression test and Begg’s rank correlation test. Egger’s test indicated possible small-study effects (p = 0.024), while Begg’s test, a more conservative method, showed no evidence of bias (p = 0.29). Given this discrepancy, we performed a trim-and-fill analysis, which suggested no studies were missing (zero studies imputed), and the adjusted pooled effect size was identical to the original estimate. Therefore, we concluded that there is no substantial evidence of publication bias or small-study effects in our meta-analysis.

### Source of heterogeneity

To identify factors associated with the size of the pooled effect for HbA1c and the amount of heterogeneity explained by some factors, we conducted subgroup analysis and meta-regression.

### Subgroup analysis

The mean effect size did not substantially vary in terms of setting, intervention modality, intervention content, strategy or duration of intervention. However, the application of behavior change theory resulted in a significant variation in the mean effect size (Q value of 5.568, df. 1, and p value 0.018) at the 0.05 level of significance. **Table 3** shows the details of the subgroup analysis.

**Table 3:** Factors associated with the size of the pooled effect of D-SMES interventions on HbA1c, a subgroup analysis using a mixed effects model (analysis).

Groups	Number of studies	Subgroup overall SMD (g) and 95% CI				Test of HO 2-tailed		Heterogeneity		
		PE	SE	Lower	Upper	Z- Value	Sig.	Q-Value	df	Sig.
Setting										
Community	4	-0.561	0.297	-1.142	-0.021	-1.888	0.059	0.618	2	0.734
Hospital	10	-0.386	0.102	-0.587	-0.186	-3.779	<0.001			
PHC	5	-0.574	0.288	-1.139	-0.009	-1.991	0.046			
Overall	19	-0.422	0.092	-0.601	-0.242	-4.603	<0.001			
Modality										
Group	15	-0.507	0.114	-0.731	-0.284	-4.453	<0.001	1.406	1	0.236
Individual	4	-0.320	0.109	-0.534	-0.107	-2.937	0.003			
Overall	19	-0.410	0.079	-0.564	-0.255	-5.201	<0.001			
Content										
Multiple	15	-0.429	0.093	-0.610	-0.247	-4.623	<0.001	0.184	1	0.668
Single	4	-0.628	0.456	-1.521	-0.265	-1.379	0.168			
Overall	19	-0.437	0.091	-0.615	-0.258	-4.508	<0.001			
Strategy										
Discrete	3	-0.341	0.137	-0.609	0.073	-2.491	0.013	0.754	1	0.385
Multifaceted	16	-0.492	0.108	-0.704	-0.280	-4.548	<0.001			
Overall	19	-0.434	0.085	-0.600	-0.268	-5.113	<0.001			
Behavior changes theory (BCT)										
No	12	-0.609	0.149	-0.900	-0.317	-4.088	<0.001	5.568	1	0.018*
Yes	7	-0.215	0.075	-0.363	-0.067	-2.851	0.004			
Overall	19	-0.295	0.067	-0.427	-0.163	-0.163	<0.001			
Duration of follow-up										
Less than 6 months	6	-0.331	0.117	-0.561	-0.101	-2.816	0.005	2.375	2	0.305
Six months	5	-0.701	0.210	-1.113	-0.290	-3.340	0.001			
Greater than 6 months	8	-0.412	0.136	-0.678	-0.147	-3.040	0.002			
Overall	19	-0.417	0.082	-0.577	-0.256	-5.094	<0.001			

*At the 0.05 sig. level, the effect size varies significantly in terms of whether BCT was applied. PE: point estimate; SE: standard error; Ho: null hypothesis.

### Meta-regression

Meta-regression was conducted to explore the effects of multiple factors (characteristics of studies) simultaneously on the pooled effect estimate and to discuss the proportion of variance explained by each factor. Adjusting for other covariates, only the application of behavior change theory showed a marginally significant association with the SMD in HbA1c (b= -0.667, 95% CI = -1.335 to 0.001, p=0.050). Accordingly, a better SMD for HbA1c was associated with D-SMES interventions not guided by BCT. **Table 4** shows the details of the meta-regression analysis. The Q statistics for the test of the model (a simultaneous test in which all coefficients, excluding intercepts, are zero) revealed that the SMD for HbA1c is not significantly explained or predicted by these covariates (Q = 7.49, df = 8, p = 0.4843). The goodness-of-fit test (where the unexplained variance is zero) shows that 17.2% of the variance is explained by the model (Tau² = 0.1724, Tau = 0.4152, I² = 84.06%, Q = 62.75, df = 10, p = <0.001).

**Table 4:** Factors associated with the size of the pooled effect of D-SMES interventions on HbA1c.

Covariate	Coefficient	SE	95% CI		Z	2-sided Sig.	Q-statistics
			Lower	Upper			
Intercept	0.533	0.977	-1.383	2.449	0.55	0.585	
Duration of follow-up							
=6 months	-0.197	0.375	-0.932	0.537	-0.53	0.598	0.51 df=2 p=0.773
>6 months	-0.230	0.335	-0.889	0.427	-0.69	0.491
<6 months	Ref.					
Application of BCT							
No	-0.667	0.341	-1.335	0.001	-1.96	0.050*	
Yes	Ref.						
Intervention Content							
Single	-0.103	0.358	-0.806	0.598	-0.29	0.772	
Multiple	Ref.						
Implementation strategy							
Multifaceted	-0.266	0.833	-1.899	1.367	-0.32	0.749	
Discrete	Ref.						
Implementation modality							
Individualized	-0.101	0.709	-1.491	1.287	-0.14	0.886	
Group	Ref.						
Intervention Setting							
Community Based	-0.482	0.326	-1.121	0.156	-1.48	0.138	Q=2.36, df=2, p**=0.307
Primary health facility	-0.239	0.353	-0.932	0.453	-0.68	0.497
Hospital outpatient clinic	Ref.						

BCT, Behavior change theory; Ref. reference group; *Marginal significance; **no significant difference across settings. Meta-regression using random effects (MM), the Z distribution, and Hedges’s g.

## Discussion

For successful target glucose levels, diabetes requires a comprehensive management plan in which patients are educated and supported to make informed decisions about diet, exercise, weight control, blood glucose monitoring, taking medication, and regular screening for complications. Current evidence on the effects of diabetes self-management education and support (D-SMES) interventions on blood glucose levels is mixed, with some studies pointing to significant glycemic benefits (11–16), whereas others have shown no significant benefits (17–19). This systematic review and meta-analysis was conducted to evaluate the effects of D-SMES interventions compared with those of usual care in improving blood glucose levels among adult patients with T2DM in the WHO African Region and to describe the core components of D-SMES interventions.

This meta-analysis revealed a significant overall effect of D-SMES interventions on HbA1c among people living with type 2 diabetes in the WHO Africa Region (SMD = -0.468 with a 95% CI of -0.658 to -0.279). The improvement in glycemic levels is considered to be clinically meaningful, as suggested by Cohen (38). The improvement in glycemic levels reported in this study is consistent with the effects reported in a previous systematic review and meta-analysis of lifestyle interventions in LMICs (39). However, this finding contrasts with the nonsignificant and inconclusive effect on HbA1c observed in a systematic review and meta-analysis of diabetes self-management interventions in Africa, where the pooled effect on HbA1c was not provided (40). This discrepancy may be partly explained by the inclusion of both type 1 and type 2 diabetes cases in (40), as well as the inclusion of countries such as Egypt, which are outside the WHO African Region. Furthermore, only two studies in that review reported a significant improvement in HbA1c.

A greater effect of D-SMES interventions on HbA1c was reported in (41), and a much weaker effect was reported in (42) were empowerment was only a measuring instrument for D-SMES interventions. The difference may be due to the difference in the number of studies included and the difference in setting. Our study is restricted to only the WHO African Region, were others were worldwide and from high income countries.

This finding has a substantial degree of heterogeneity (I^2^ = 85.55%, Tau squared = 0.137), which tells us that some 85.5% of the variance reflects variance in true effects rather than sampling error. If we assume that the true effects are normally distributed, we would expect that in some 95% of all populations comparable to those in the analysis, the true effect size will fall in the range of -1.27 to 0.34 (prediction interval), which shows that in some populations, D-SMES intervention has a large clinical effect, whereas in others, the effect is small. The results were not overly influenced by any one study, since the basic conclusion remained unchanged, with any one study removed from the sensitivity analysis. No small study effect was shown in this meta-analysis; this may be due to the authors of randomized trials, who are likely to want to see RCTs published even if the result is negative because of the effort involved.

In the current meta-analysis, a subgroup analysis was conducted on the basis of the setting, intervention modality, intervention content, intervention strategy, application of behavior change theory, and duration of follow-up. Concerning the setting where the intervention was conducted, this meta-analysis showed a significant pooled estimate in all settings (community, hospital outpatient clinic, or primary healthcare facilities), but the SMD did not substantially vary across settings. This may be due to the small number of studies that were included in each setting. However, a review conducted in LMICs (39) showed that lifestyle interventions delivered by healthcare professionals in hospital or clinic settings were deemed most effective. Concerning the intervention modality, we found that the majority of the interventions were delivered in group settings. Group-based education has been found to be significantly more effective than individualized educational interventions. However, the variation was not significant between the group based and individual-based interventions. This is consistent with the finding that group-based education has become the preferred format for delivering self-management education (43). However, intervention modalities should be tailored to individual preferences and learning styles since people with diabetes have different learning needs (44).

With respect to the content of interventions, there was no substantial difference between interventions with multiple or single contents. One possible explanation could be that multiple behavioral interventions can be burdensome and complex for patients and that long-term interventions are needed to become habitual. Inconsistent findings have been reported (39), where those that included multiple education components (e.g., diet, physical activity, taking medication, smoking cessation) were deemed most effective.

With respect to the type of implementation strategy, multifaceted interventions were found to have a substantial effect on HbA1c compared with interventions with discrete implementation strategies. However, the overall pooled estimate does not vary based on the type of implementation strategy.

Interventions designed to influence diabetes self-management behavior are more likely to be beneficial when they are grounded in theories. However, the current meta-analysis indicated that interventions grounded in theory had a nonsignificant effect on HbAlc compared with interventions not guided by behavior change theories. This may be related to only seven out of the 19 studies in this review that mentioned the name of behavioral change theories. This might also be related to the fact that other studies used a theoretical model but did not report that; it also raises the question about the usefulness of such models.

Even though the effect was not significant according to the duration of follow-up, average durations of interventions (six months) were more likely to have better effect on reduction of HbA1c levels. In contrast, other reviews (41, 45) have shown that short educational interventions (less than 6 months) are better than longer interventions. One possible explanation may be associated with the initial motivation of the participant to be empowered to obtain positive results in a short period of time. The duration of contact hours between the intervention provider and patient may have contributed to this difference. Another explanation is the difference in the quality (fidelity) of interventions. In addition, relapses in behavior are expected among some of the participants.

The strengths of this review include the use of a registered protocol and a comprehensive search strategy in multiple databases; only RCTs were included, and the methodological quality of the majority of the included studies was high. This study also has limitations. First, studies published in the English language were only considered for this systematic review. Second, significant heterogeneity was observed across studies. Third, the inclusion of only 19 studies in the review is an indication that the conclusions drawn are based on limited data. Despite these limitations, we believe that this SRMA provides useful information that may inform the implementation of D-SMES interventions in Africa and other developing countries.

## Conclusion

Diabetes self-management education and support interventions are moderately effective in controlling blood glucose levels in T2DM patients within the WHO African Region. The majority of the interventions had statistically significant positive effects on HbA1c. Few studies on D-SMES have been conducted in the WHO African Region. Therefore, the need to scale up interventional studies on D-SMES in the region is of paramount importance. Moreover, the usefulness and appropriate use of behavior change theories should be investigated in D-SMES interventions.

## Data Availability

The original contributions presented in the study are included in the article/**Supplementary Material**. Further inquiries can be directed to the corresponding author.

## Supplementary material

Additional File 1 PRISMA 2020 Checklist.

Additional File 2 Data extracted from the included studies.

Additional file 3 Characteristics of D-SMES interventions of the included studies.

Additional File 4 RoB 2 quality assessment of the included studies.
