# Supplementary material for: Effectiveness of diabetes self-management education and support interventions on glycemic levels among people living with type 2 diabetes in the WHO African Region: a Systematic Review and meta-analysis
Source: Front Clin Diabetes Healthc. 2025 Jun 3;6:1554524. doi: 10.3389/fcdhc.2025.1554524 (PMC12170312; doi:10.3389/fcdhc.2025.1554524)
Supplement: Additional file 3 — Characteristics of D-SMES interventions of the included studies. [file Table3.docx]

| Study Name | Modality | Provider | Intervention component | Implementation Strategy | BCT | Intervention description |
| --- | --- | --- | --- | --- | --- | --- |
| Assah et al., 2015 | Group | Peer Educators  /Supporter | Multiple | Multifaceted | No | Monthly six group meetings, personal encounters between peer supporters and their group members, and telephone calls. |
| Debussche et al., 2018 | Group | Peer Educators  /Supporter | Multiple | Multifaceted | Yes | Culturally tailored structured patient education in 3 courses of 4 sessions (177*1.5hr) and (implementation of curricula and the distribution of hand-outs. |
| Essien et al., 2021 | Group | Health Care Providers | Multiple | Multifaceted | No | 12 structured teaching sessions lasting around two hours each, comprised of lectures and group discussions, the sessions were interactive and included generic diabetes education videos, and leaflets were also provided |
| Gathu et al. 2018 | Individual | Diabetes Educator | Multiple | Multifaceted | Yes | Three one-hour sessions after every six weeks + patient guide in the form of diabetes booklet and graphic material + telephone reminders on behavioural assessment, goal-setting and problem solving. |
| Hailu et al., 2018 | Group | Health Care Providers | Multiple | Multifaceted | No | Six DSME sessions for 1.5 h every month for 6 consecutive months comprised of brief education, discussion, experience sharing, reminded of upcoming sessions by phone take-home activities, conclusion and revision. Supported with illustrative pictures handbooks and fliers |
| Huimin et al., 2014 | Individual | Research team | Single | Discrete | No | The LEX group underwent supervised exercises at 50%VO2 peak for 45min/session, 3–5 times/week, and the VEX group exercised under supervision at 75% VO2 peak for 45min/session, 3 times/week. |
| Muchiri et al., 2015 | Group | Dietician | Single | Multifaceted | No | Eight weekly (2–2·5 h) group nutrition education sessions and follow-up sessions. The NE programme consisted of three components: (i) the curriculum (eight weekly sessions, 2 to 2.5 h each); (ii) follow-up sessions (four monthly meetings and two bimonthly meetings each lasting 1.5 h); and (iii) vegetable gardening (demonstration of sowing/transplantation of vegetables). |
| Muchiri et al., 2021 | Group | Dietician | Single | Multifaceted | Yes | Seven-monthly group education sessions, bi-monthly follow-up sessions, 15-minute individual session, workbook + education materials |
| Ojieabu et al., 2017 | Individual | Pharmacist | Multiple | Discrete | No | A four-session pharmacist education on diabetes and hypertension, their complications, risks, preventive measures and management. In particular, they were counselled on the need for medication and treatment adherence such as clinic visits and lifestyle modifications including diet and exercise. |
| Mash et al., 2014 | Group | Health promoter | Multiple | Multifaceted | Yes | Four 60 minutes group education that focused on understanding diabetes, living a healthy lifestyle, understanding the medication and avoiding complications |
| Ng’ang’a et al., 2022 | Group | Health Care Providers | Single | Multifaceted | No | Participants received a glucose meter, blood test strips, logbook, waste management box and training on how to conduct SMBG in addition to usual care and monthly follow-up. |
| Asante et al, 2020 | Individual | Peer Educators  /Supporter | Multiple | Discrete | No | A total of 12 weeks of mobile phone follow-up calls by a nurse with a mean duration of 12 minutes each (2 calls per week for the first 4 weeks, followed by a weekly call for the following 8 weeks, totalling 16 calls). The content of the calls thus included information on diet, exercise, medication taking, self-monitoring of blood glucose, and foot care, Additionally, individualized self-management goals were evaluated. |
| Agatha et al., 2010 | Group | Research team | Multiple | Multifaceted | Yes | A daily walk and diet education intervention program, After the four weekly group sessions, participants continued at home from week five of the program. They received motivating text messages fortnightly. |
| Amendezo et al., 2017 | Group | Health Care Providers | Multiple | Multifaceted | No | Monthly 45 minutes counselling and education session plus educational pamphlets |
| David EA et al., 2021 | Group | Research team | Multiple | Multifaceted | No | two 30–45-minute face-to-face sessions with the lead researcher at baseline and month three, an educational package, and follow-up calls or texts every six weeks for session review |
| Farmer et al., 2021 | Individualized | Research team | Multiple | Multifaceted | Yes | motivational and educational text-messages sent to participants on different days, three to four times weekly over a period of 12 months. |
| Lamptey R et al., 2023 | Group | Health Care Providers | Multiple | Multifaceted | No | One session of structured DSME, delivered by two educators to groups of six to ten participants in |
| Thuita et al., 2020 | Group | Research team (PI) | Multiple | Multifaceted | No | one day, over 6 h" |
| Diriba DC et al., 2023 | Group | Research team | Multiple | Multifaceted | Yes | a nutrition education programme with peer-to-peer support (NEP) for 2 h per week for 8 weeks and weekly peer-to-peer interactions for 8 weeks |
