# Supplementary material for: Effectiveness of diabetes self-management education and support interventions on glycemic levels among people living with type 2 diabetes in the WHO African Region: a Systematic Review and meta-analysis
Source: Front Clin Diabetes Healthc. 2025 Jun 3;6:1554524. doi: 10.3389/fcdhc.2025.1554524 (PMC12170312; doi:10.3389/fcdhc.2025.1554524)
Supplement: Additional File 4 — RoB 2 quality assessment of the included studies. [file Table4.docx]

| **Signaling questions** | **Response options** | Assah et al., 2015 | Debussche et al., 2018 | Essien et al., 2021 | Gathu et al. 2018 | Hailu et al., 2018 | Huimin et al., 2014 | Muchiri et al., 2015 | Muchiri et al., 2021 | Ojieabu et al., 2017 | Mash et al., 2014 | Ng’ang’a et al., 2022 | Asante et al, 2020 | Agatha et al., 2010 | Amendezo et al., 2017 | David EA et al., 2021 | Farmer et al., 2021 | Lamptey R et al., 2023 | Thuita et al., 2020 | Diriba DC et al., 2023 |
| --- | --- | --- | --- | --- | --- | --- | --- | --- | --- | --- | --- | --- | --- | --- | --- | --- | --- | --- | --- | --- |
| Domain 1: Risk of bias arising from the randomization process | |  |  |  |  |  |  |  |  |  |  |  |  |  |  |  |  |  |  |  |
| 1.1 Was the allocation sequence random? | Y / PY / PN / N / NI | Y | Y | Y | Y | Y | Y | Y | Y | Y | Y | Y | Y | Y | Y | Y | Y | Y | Y | Y |
| 1.2 Was the allocation sequence concealed until participants were enrolled and assigned to interventions? | Y / PY / PN / N / NI | N | Y | Y | Y | PY | PY | Y | Y | PN | Y | Y | Y | Y | Y | Y | Y | Y | Y | Y |
| 1.3 Did baseline differences between intervention groups suggest a problem with the randomization process? | Y / PY / PN / N / NI | N | N | N | N | N | N | N | Y | N | N | N | N | N | Y | Y | Y | Y | Y | Y |
| Risk-of-bias judgments | Low / High / Some concerns | High | Low | Low | Low | Low | Low | LOW | LOW | some con | LOW | Low | LOW | LOW | LOW | LOW | LOW | LOW | LOW | LOW |
| Domain 2: Risk of bias due to deviations from the intended interventions (effect of assignment to intervention) | |  |  |  |  |  |  |  |  |  |  |  |  |  |  |  |  |  |  |  |
| 2.1. Were participants aware of their assigned intervention during the trial? | Y / PY / PN / N / NI | Y | Y | Y | Y | Y | Y | Y | Y | Y | Y | Y | N | N | Y | Y | Y | Y | Y | Y |
| 2.2. Were careers and people delivering the interventions aware of participants' assigned intervention during the trial? | Y / PY / PN / N / NI | Y | Y | Y | Y | Y | Y | Y | Y | Y | Y | Y | N | N | Y | Y | Y | Y | Y | Y |
| 2.3. If Y/PY/NI to 2.1 or 2.2: Were there deviations from the intended intervention that arose because of the trial context? | NA / Y / PY / PN / N / NI | N | N | Y | N | N | N | N | N | N | N | N |  |  | N | N | N | N | N | N |
| 2.4 If Y/PY to 2.3: Were these deviations likely to have affected the outcome? | NA / Y / PY / PN / N / NI |  |  | N |  |  | N |  |  |  |  |  |  |  |  |  |  |  |  |  |
| 2.5. If Y/PY/NI to 2.4: Were these deviations from intended intervention balanced between groups? | NA / Y / PY / PN / N / NI |  |  |  |  |  |  |  |  |  |  |  |  |  |  |  |  |  |  |  |
| 2.6 Was an appropriate analysis used to estimate the effect of assignment to intervention? | Y / PY / PN / N / NI | Y | Y | Y | Y | Y | Y | Y | Y | Y | Y | Y | Y | Y | Y | Y | Y | Y | Y | Y |
| 2.7 If N/PN/NI to 2.6: Was there potential for a substantial impact (on the result) of the failure to analyze participants in the group to which they were randomized? | NA / Y / PY / PN / N / NI |  |  |  |  |  |  |  |  |  |  |  |  |  |  |  |  |  |  |  |
| Risk-of-bias judgment | Low / High / Some concerns | some concerns | Low | Low | Low | Low | Low | LOW | LOW | LOW | LOW | LOW | LOW | LOW | LOW | LOW | LOW | LOW | LOW | LOW |
| Domain 3: Missing outcome data |  |  |  |  |  |  |  |  |  |  |  |  |  |  |  |  |  |  |  |  |
| 3.1 Were data for this outcome available for all, or nearly all, participants randomized? | Y / PY / PN / N / NI | Y | Y | N | N | N | N | Y | Y | N | N | Y | Y | N | N | Y | N | Y | N | Y |
| 3.2 If N/PN/NI to 3.1: Is there evidence that the result was not biased by missing outcome data? | NA / Y / PY / PN / N |  |  | Y | N | Y | N |  |  | PY | Y |  |  | Y | Y |  | Y |  | Y |  |
| 3.3 If N/PN to 3.2: Could missingness in the outcome depend on its true value? | NA / Y / PY / PN / N / NI |  |  |  | PN |  | N |  |  | N |  |  |  |  |  |  |  |  |  |  |
| 3.4 If Y/PY/NI to 3.3: Is it likely that missingness in the outcome depended on its true value? | NA / Y / PY / PN / N / NI |  |  |  |  |  |  |  |  |  |  |  |  |  |  |  |  |  |  |  |
| Risk-of-bias judgment | Low / High / Some concerns | Low | Low | Low | Low | Low | Low | low | LOW | some con | LOW | LOW | LOW | LOW | LOW | LOW | LOW | LOW | LOW | LOW |
| Domain 4: Risk of bias in measurement of the outcome | |  |  |  |  |  |  |  |  |  |  |  |  |  |  |  |  |  |  |  |
| 4.1 Was the method of measuring the outcome inappropriate? | Y / PY / PN / N / NI | N | N | N | N | N | N | N | N | Y | N | N | N | N | N | N | N | N | N | Y |
| 4.2 Could measurement or ascertainment of the outcome have differed between intervention groups? | Y / PY / PN / N / NI | N | N | N | N | N | N | N | N | N | N | N | N | N | N | N | N | N | N | N |
| 4.3 If N/PN/NI to 4.1 and 4.2: Were outcome assessors aware of the intervention received by study participants? | NA / Y / PY / PN / N / NI | PN | PY | N | N | N | Y | N | N | Y | Y | PY | N | N | Y | N | N | N | N | N |
| 4.4 If Y/PY/NI to 4.3: Could assessment of the outcome have been influenced by knowledge of intervention received? | NA / Y / PY / PN / N / NI | PN | PN |  |  |  | N |  |  | N | N | N |  | N | PN |  |  |  |  |  |
| 4.5 If Y/PY/NI to 4.4: Is it likely that assessment of the outcome was influenced by knowledge of intervention received? | NA / Y / PY / PN / N / NI | PN |  |  |  |  |  |  |  |  |  |  |  |  |  |  |  |  |  |  |
| Risk-of-bias judgement | Low / High / Some concerns | Low | Low | Low | Low | Low | Some con | LOW | LOW | HIGH | some con | LOW | LOW | LOW | LOW | LOW | LOW | LOW | LOW |  |
| Domain 5: Risk of bias in selection of the reported result |  |  |  |  |  |  |  |  |  |  |  |  |  |  |  |  |  |  |  |  |
| 5.1 Were the data that produced this result analysed in accordance with a pre-specified analysis plan that was finalized before un-blinded outcome data were available for analysis? | Y / PY / PN / N / NI | Y | Y | PY | Y | Y | Y | Y | Y | Y | y | Y | Y | Y | Y | Y | Y | Y | Y | Y |
| 5.2. ... multiple eligible outcome measurements (e.g. scales, definitions, time points) within the outcome domain? | Y / PY / PN / N / NI | N | N | N | N | N | N | N | N | N | n | N | N | N | N | N | N | N | N | N |
| 5.3 ... multiple eligible analyses of the data? | Y / PY / PN / N / NI | N | N | N | N | N | N | N | N | N | n | N | N | N | N | N | N | N | N | N |
| Risk-of-bias judgment | Low / High / Some concerns | Low | Low | Low | Low | Low | LOW | LOW | LOW | LOW | low | LOW | LOW | LOW | LOW | LOW | LOW | LOW | LOW | LOW |
| Overall risk of bias |  |  |  |  |  |  |  |  |  |  |  |  |  |  |  |  |  |  |  |  |
| Risk-of-bias judgment | Low / High / Some concerns | High | Low | Low | Low | Low | some con | Low | Low | HIGH | some con | Low | Low | Low | Low | Low | Low | Low | Low | LOW |

Y: Yes; PN: Probably Yes; N: No; PN: Probably No; NI: Not Applicable
